# Optical Fiber Demodulation System with High Performance for Assessing Fretting Damage of Steam Generator Tubes

**DOI:** 10.3390/s18010201

**Published:** 2018-01-12

**Authors:** Peijian Huang, Ning Wang, Junying Li, Yong Zhu, Jie Zhang, Zhide Xi

**Affiliations:** 1 The Key Laboratory of Optoelectronic Technology & System (Ministry of Education), Chongqing University, Chongqing 400044, China; 20113269@cqu.edu.cn (P.H.); junyingli@cqu.edu.cn (J.L.); yongzhu@cqu.edu.cn (Y.Z.); zhangjie@cqu.edu.cn (J.Z.); 2 Nuclear Power Institute of China, Chengdu 610041, China; xizhide@sina.com

**Keywords:** optical fiber, fiber Fabry-Perot sensor, steam generator tube, F-P signal demodulation

## Abstract

In order to access the fretting damage of the steam generator tube (SGT), a fast fiber Fabry-Perot (F-P) non-scanning correlation demodulation system based on a super luminescent light emitting diode (SLED) was performed. By demodulating the light signal coming out from the F-P force sensor, the radial collision force between the SGT and the tube support plate (TSP) was interrogated. For higher demodulation accuracy, the effects of the center wavelength, bandwidth, and spectrum noise of SLED were discussed in detail. Specially, a piezoelectric ceramic transducer (PZT) modulation method was developed to get rid of the interference of mode coupling induced by different types of fiber optics in the demodulation system. The reflectivity of optical wedge and F-P sensor was optimized. Finally, the demodulation system worked well in a 1:1 steam generator test loop and successfully demodulated a force signal of 32 N with a collision time of 2 ms.

## 1. Introduction

The faults related to the steam generator tube (SGT) rupture accounts for 75% of all failure risks in pressurized water reactor nuclear power plants [1]. As the main cause of SGT rupture, fretting damage has been addressed as a worldwide problem in the safety of nuclear power plants for a long time [2,3,4,5,6]. The SGT is the boundary between the reactor cooling system and the secondary circuit and suffers from the fretting damage caused by fluid motion and thermal energy transmission (350 °C, 15 MPa, humid). After long-term running, its special working conditions can cause fatigue cracks and premature failure of the SGT. Therefore, a reasonable assessment of SGT fretting damage has an important value in the structure design, optimization, and service life prediction of the SGT.

The fretting work is an important parameter to assess the extent of fretting damage of the SGT. This parameter, *W*, was commonly used to relate the energy dissipation under sliding/impact fretting wear conditions to the volumetric wear losses, which is given by [5].

(1) $$W=\frac{\int_{0}^{\tau }\left|F\left(\tau \right)\right|D_{s}\left(\tau \right)d\tau }{\int_{0}^{\tau }d\tau },$$

where |*F*| is the radial collision force, *D_s_* is the distance of axial displacement, and *τ* is the time over which the work rate is averaged. The radial collision force, the impact time, and the axial displacement are important parameters to calculate the fretting work.

**Figure 1:**
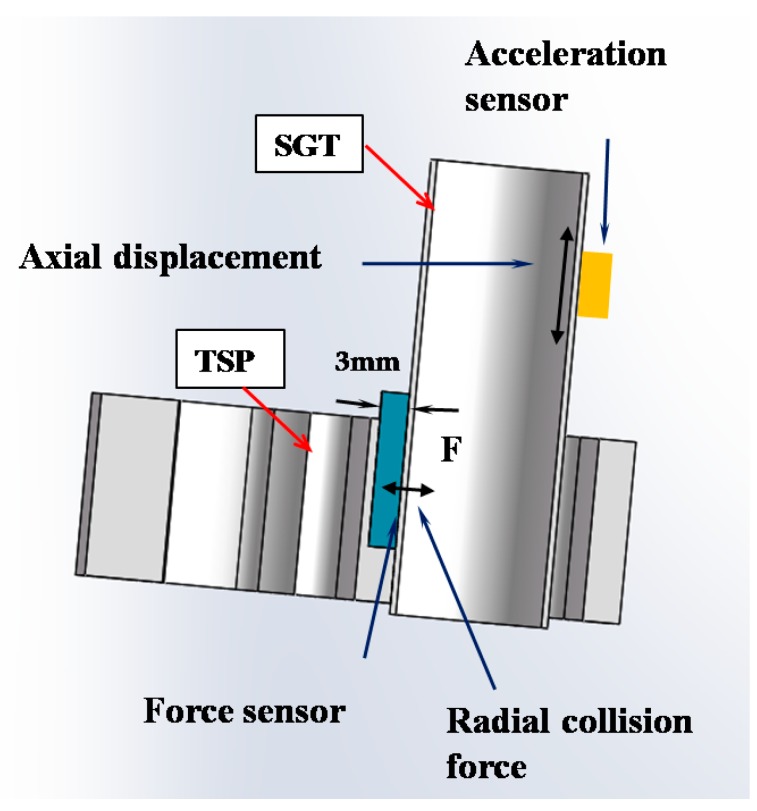
The sectional view of the steam generator tube (SGT) and the tube support plate (TSP).

As shown in Figure 1, the narrow space in the contact position between the SGT and tube support plate (TSP) (about 4 mm) is not large enough to install the traditional electric force sensors. Compared with the electric force sensors, the fiber sensors have outstanding advantages due to their small size and anti-electromagnetic interference [7]. As for the high requirements of the radial collision force measurement, we have developed a novel optical Fabry-Perot (F-P) force sensor [8], with the advantages of small volume (17 × 5 × 3 mm^3^) and high performance in a humid, high pressure, and vibrating working environment. In our F-P force sensor, the F-P cavity length variation is proportional to the magnitude of the applied force.

In order to obtain the F-P cavity length (for calculating the radial collision force) and the collision time, a corresponding high-speed optical demodulation system is extremely necessary. The first question is how to demodulate the light signal coming from the F-P force sensor and to obtain the F-P cavity length variation. Due to the short impact time of radial collision force [6], the second question is how to achieve a fast demodulation speed (above 5 KHz). The third question is how to obtain a long demodulation range of F-P cavity length variation (0–25 μm, a corresponding force of 0–200 N).

The demodulation methods of fiber F-P cavity length can be divided into two types: the intensity demodulation method and the phase demodulation method [7]. Hu developed a novel fiber optic Fabry-Perot current sensor based on magnetic fluid as the medium in F-P interference cavity, and a demodulation method based on slanted fiber Bragg grating (FBG) wavelength measurement system [9]. Ge described a novel optical fiber sensor based on a Fabry-Perot interferometer and a phase demodulation method based on Fourier transformation [10]. Lu combined the subcarrier technology with a dual-wavelength demodulation method to track the cavity length variation of a micro fiber-optic F-P [11]. Ran developed a novel miniature fiber-optic F-P refractive-index sensor based on the intensity demodulation method [12]. These demodulation methods are insufficient to meet the requirements (range of 0–25 μm, demodulation speed above 5 KHz) at the same time. However, their counterpart, the non-scanning correlation demodulation method that lacks moving parts and an expensive laser as the light source, is capable of realizing large cavity length measurement [13].

In this paper, targeting the questions mentioned above, we developed a novel fast fiber optical F-P non-scanning correlation demodulation system based on the super luminescent light emitting diode (SLED). Its key parameter effects and optimized design are discussed theoretically and experimentally. The results meet the requirements of the cavity length variation range (0–25 μm) and a demodulation speed (5 KHz). Especially, our system, with high performance, was proved on the 1:1 steam generator test loop (which simulated a real steam generator with the same geometry and system design).

## 2. Theory and Simulation of the Modulation System

### 2.1. The Principle of the Non-Scanning Correlation Demodulation System

**Figure 2:**
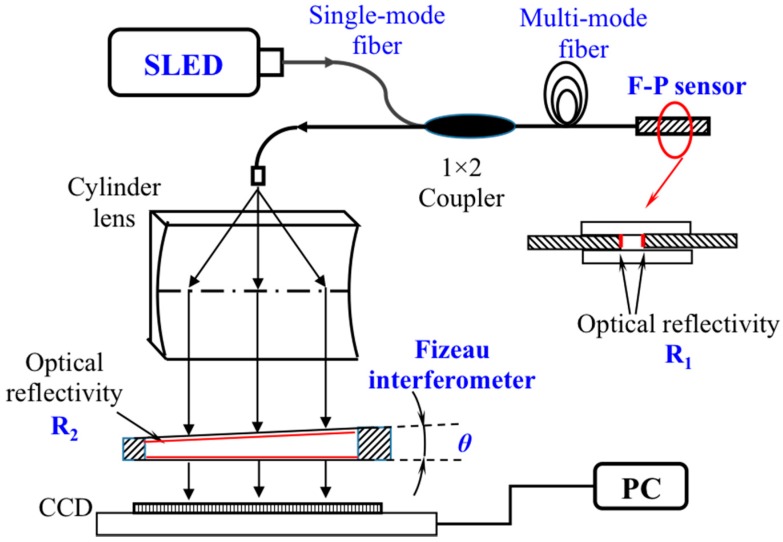
A schematic of the optical Fabry-Perot (F-P) non-scanning correlation demodulation system. (SLED, super luminescent light emitting diode; CCD, charge coupled devices; PC, personal computer).

The whole sensing system is shown in Figure 2, consisting of a fast charge coupled device (CCD), an SLED light source (with high power density, for shorter CCD integration time and higher demodulation speed of up to 5–10 kHz), a single-mode (SM) fiber (for the SLED light source coupling), a 1 × 2 fiber coupler, an F-P force sensor built by a multi-mode (MM) fiber (for high collection efficiency of F-P cavity information), a cylinder lens (for radiating the light signal from fiber), a Fizeau interferometer (also called optical wedge), and a personal computer (PC). Note that, due to the small diameter of the SLED’s effective emission area (about 4~5 μm), a SM fiber with a core diameter of 4 μm is used for a high coupling efficiency. In the measuring process, the F-P force sensor is used to convert the applied force into a light signal that carries the information of the F-P cavity length. Then, the light signal is radiated on the upper surface of the optical wedge by the cylindrical lens and converted into an electric signal by the linear CCD.

The incident light on the CCD is the SLED spectrum modulated by the F-P sensor and the Fizeau interferometer, described by formula (2) [13].

(2) $$I_{out}\left(x\right)=\int_{\lambda_{\min}}^{\lambda_{\max}}\frac{2R_{1}\left(1+\cos\frac{4\pi L}{\lambda }\right)}{1+R_{1}^{2}+2R_{1}\cos\frac{4\pi L}{\lambda }}\times \frac{{\left(1-R_{2}\right)}^{2}}{1+R_{2}^{2}-2R_{2}\cos\left(\frac{4\pi x\tan\theta }{\lambda }\right)}e^{-\frac{{\left(\lambda -\lambda_{p}\right)}^{2}}{B_{\lambda }^{2}}}I_{0}d\lambda ,$$

where *R_1_* is the end face reflectivity of the fiber optic F-P sensor, *R_2_* is the reflectivity of the Fizeau interferometer, *B_λ_* is the bandwidth of the SLED spectrum, *λ_p_* is the central wavelength of the SLED spectrum, *λ_min_*~*λ_max_* is the wavelength range of SLED, *θ* is the optical wedge angle, *L* is the F-P cavity length, *x* is the optical wedge length and also the photoreceptor length of CCD, *I_0_* is the input light intensity from SLED, and $\exp(-{\left(\lambda -\lambda_{p}\right)}^{2}/B_{\lambda }^{2})$ results from the Gaussian of SLED spectrum.

**Figure 3:**
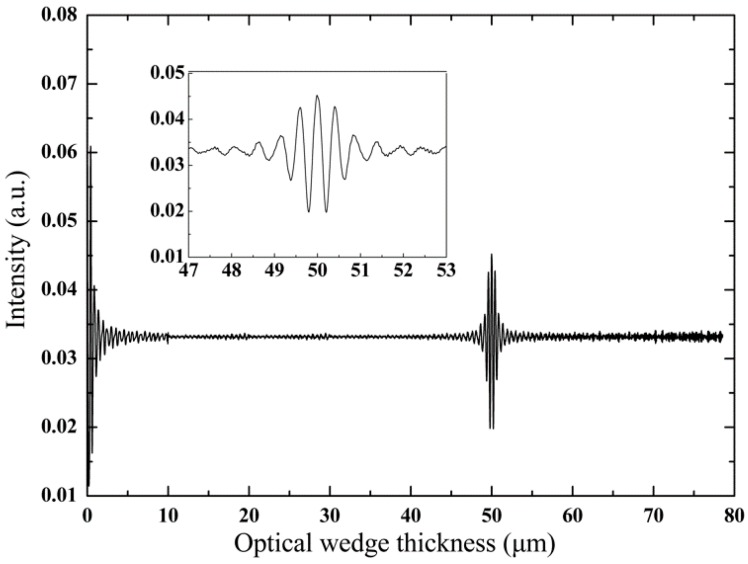
The relationship between the optical wedge thickness and the light intensity output.

In order to see the intensity of the output light clearly, we present the simulation result in Figure 3, under the parameter conditions of *R*_1_ = 0.2, *R*_2_ = 0.5, *L* = 50 μm, *θ* = 0.05°, *λ_p_* = 840 nm, and *B_λ_* = 200 nm.

It can be seen that the maximum intensity of the correlation signal appears when the thickness of the optical wedge meets 50 μm, and this value equals to the F-P cavity length. Therefore, the F-P cavity length could be obtained by determining the CCD pixel position of the peak intensity signal.

As for this system, there are several points to be mentioned specially. (1) How does the characteristics of SLED affect the demodulation signal? (2) There are two interference devices, the F-P cavity (in force sensor) and the optical wedge. How to choose the right parameters of two devices to obtain a demodulation signal with high performance? (3) A single-mode fiber is used to collect the SLED light to gain high power density, while a multi-mode fiber is used to couple the F-P cavity information. How to eliminate the influence of mode-mode interference in the demodulation system? In Section 3, we will discuss them one by one.

### 2.2. The Principle of the F-P Force Sensor

**Figure 4:**
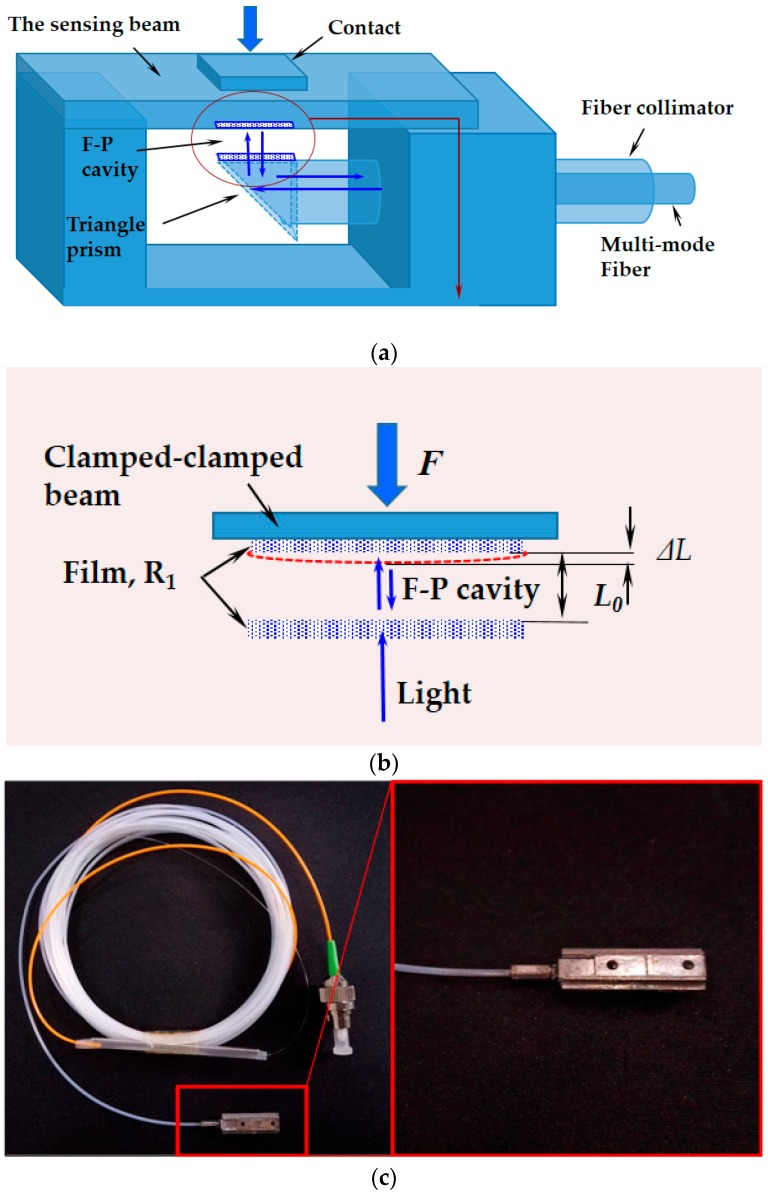
(**a**) Three-dimensional structure of our Fabry-Perot (F-P) force sensors with clamped-clamped beam element; (**b**) a simplified structure of the F-P cavity length change induced by the force F for a clamped-clamped beam; (**c**) the encapsulated F-P force sensor with the overall dimensions of 17 mm × 5 mm × 3 mm (L × W × H).

Due to the small gap at the contact position (less than 4 mm) between the SGT and the TSP, a 45° reflection prism, a fiber collimator, and clamped-clamped beam structure were utilized in the F-P force sensor. As shown in Figure 4a–c, the beam deflections of clamped-clamped beam equal to the change of the F-P cavity length. The F-P cavity length changes when a force is exerted on the force-sensitive element (sensing beam) [8].

The cavity length variation is related to the magnitude of the applied force *F*. The non-scanning correlation demodulation method is used to obtain the F-P cavity length variation induced by the force *F*. The relationship between the F-P cavity length variation and the applied force *F* in two different sensing structures is given by

(3) $$F_{1}=\Delta L\frac{{16Ebh}^{3}}{l^{3}}=\left(L_{0}-{L_{0}}^{\prime }\right)\frac{{16Ebh}^{3}}{l^{3}}$$

where *F*_1_ is the force exerted on the clamped-clamped beam, *l* is the beam length, *h* is the beam thickness, *b* is the beam width, *L*_0_ is the original F-P cavity length, *L*_0_′ is the F-P cavity length induced by the force, *ΔL* is the F-P cavity length variation, and *R*_1_ is the reflective index.

Therefore, in the F-P force sensor, the F-P cavity length variation caused by the radial collision force of SGT can be obtained by the demodulation system.

## 3. The Parameters Calculation of Non-Scanning Demodulation System

### 3.1. Effects of Light Source

#### 3.1.1. Effects of Wavelength Width

**Figure 5:**
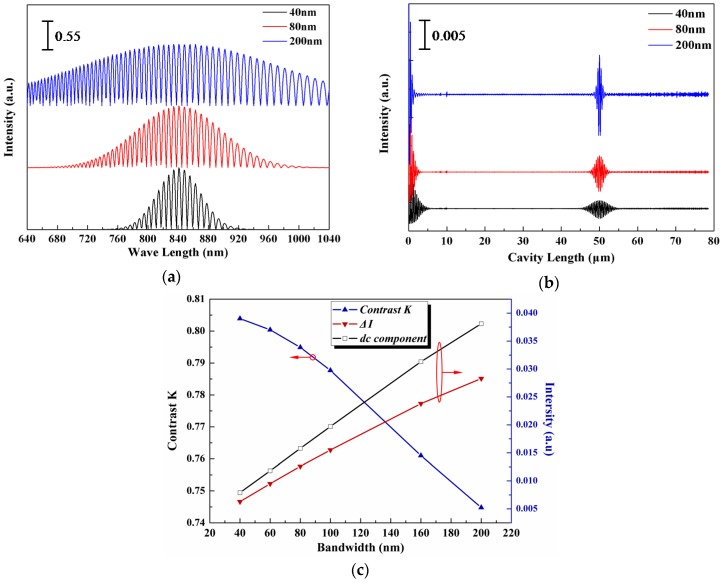
The simulated signal with different bandwidth of 40 nm, 80 nm and 160 nm: (**a**) the F-P reflective light intensity, and (**b**) the demodulation signals; (**c**) the contrast K, ΔI, *dc* component at different bandwidth of SLED.

The output of the F-P cavity is influenced by the bandwidth of SLED. The simulation result is shown in Figure 5a, under the simulation conditions of *R*_1_ = 0.2, *R*_2_ = 0.5, *L* = 50 μm, *θ* = 0.05°, and *λ_p_* = 840 nm. The corresponding intensity of the light coming from the optical wedge is shown in Figure 5b. As shown in Figure 5c, three parameters are used to evaluate the demodulation quality, *ΔI* is the difference of the maximum intensity *I_max_* and the minimum intensity *I_min_*, *dc* component is the averaged value of the intensity *I_out_*, and the signal contrast *K* is the ratio of F-P cavity length signal intensity (*I_max_*−*I_min_*) and *dc* component. We can discuss as follows.

(1) With the increase of SLED bandwidth, the spectrum of the F-P cavity was widened.

(2) At the demodulation position (~50 μm), with the increase of SLED bandwidth, the *dc* value increased, the signal contrast *K* reduced, and the demodulation signal width from the optical wedge became narrower.

(3) A signal with larger signal strength and higher contrast is preferable in CCD detection and subsequent circuit processing. Therefore, a SLED with a larger bandwidth has a better performance in the demodulation system.

#### 3.1.2. Effects of Central Wavelength

**Figure 6:**
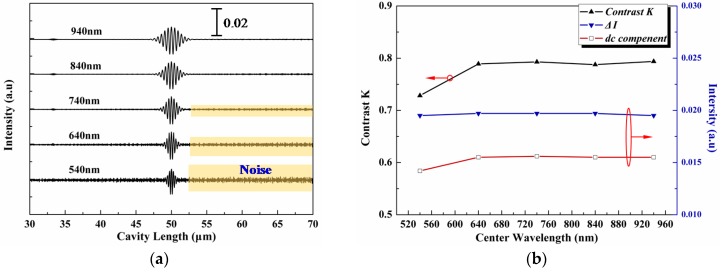
The simulation result of different central wavelength at the same bandwidth 200 nm, (**a**) the demodulation intensity, (**b**) the corresponding contrast *K*, intensity *dc*, and *ΔI*.

With the same simulation method, the simulation result of different central wavelength at the same bandwidth 200 nm is shown in Figure 6a,b. We analyzed as follows.

(1) The correlation demodulation signals became sharper and narrower with the blue shift of SLED center wavelength of the SLED, while there was no obvious change of the parameters of *ΔI*, *dc* component, and the contrast *K*.

(2) When the center wavelength was short than 740 nm, the strong noise appeared at the position of optical wedge thickness over 60 μm. This noise signal would gradually superimpose on the effective correlated demodulation signal, leading to a deterioration of the demodulation accuracy.

(3) Taking a comprehensive consideration on the theoretical analyses and commercial SLED products, a SLED with the center wavelength of 840 nm and the bandwidth of 50 nm was employed in the demodulation system.

### 3.2. Reflectivity of F-P Sensor and Optical Wedge

The light signal is modulated by two interference devices, the F-P cavity (in the force sensor) and the optical wedge. The reflectivity of the two fiber end faces of the F-P cavity and the two surfaces of the optical wedge should be considered in our design. 

**Figure 7:**
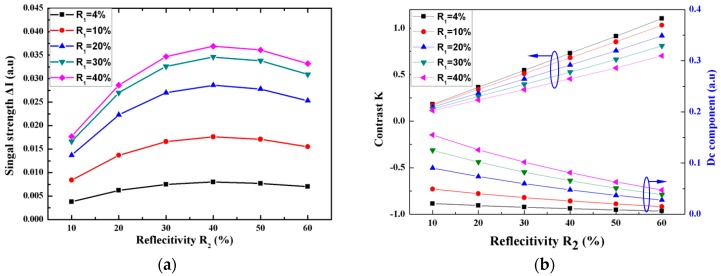
The key evaluation parameters simulated in different reflectivity R_1_ and R_2_: (**a**) *ΔI*, (**b**) *dc* component and contrast *K*.

(1) In order to gain an F-P interference signal with high quality, the reflectivity of the two ends of F-P cavity should be equivalent. Similarly, an equivalent reflectivity of the two inner faces of the optical wedge is adopted.

(2) We simulated the signal strength *ΔI* from the optical wedge using the different reflectivity of the F-P cavity (*R_1_*) and optical wedge (*R_2_*), shown in Figure 7a. The *dc* component and contrast *K* of corresponding signal is shown in Figure 7b.

(3) When the reflectivity *R*_1_ was fixed, *ΔI* first increased and then decreased using the increase of reflectivity *R*_2_. When *R*_2_ was set to 40%, *ΔI* had the largest value. The *dc* component of the signal reduced with the increase of *R*_1_ for a fixed value of *R*_2_. The contrast *K* increased with the increase of *R*_2_. Therefore, CCD detection and processing circuit, the reflectivity of *R*_1_ and *R*_2_ was selected as 20% and 40%, respectively.

### 3.3. Effects of Mode-Mode Interference

**Figure 8:**
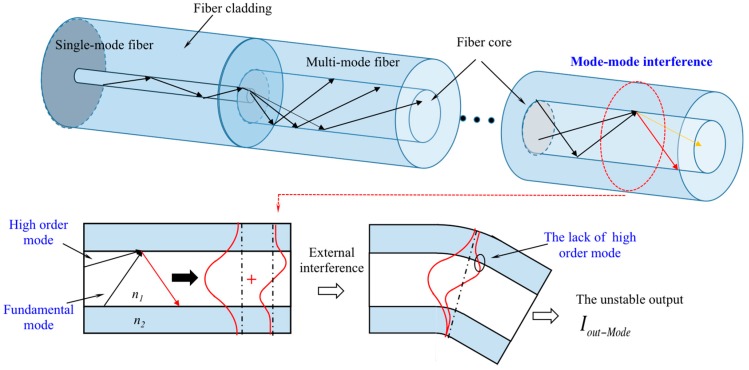
Schematic of single-mode (SM)–multi-mode (MM) fiber coupling in the optical system.

As we mentioned in Section 2, the mode-mode interference occurred because of the fiber mode mismatch, shown in Figure 8 [14]. When the light energy coming out from a SM fiber is coupled into a MM fiber, higher-order modes of MM fiber are excited and interfered with each other.

The spectral distribution of SLED output affected by the mode-mode interference is given by

(4) $$I_{out-Mode}=\int_{\lambda_{\min}}^{\lambda_{\max}}\left[\sum_{i=1}^{N}\eta_{i}^{2}+\sum_{i\neq j=1}^{N}\eta_{i}\eta_{j}\Delta p\;\cos\left[\left(\beta_{i}-\beta_{j}\right)L\right]\right]\times I_{0}e^{-\frac{{\left(\lambda -\lambda_{p}\right)}^{2}}{B_{\lambda }^{2}}},$$

where *η_i_* is the intensity coefficients assigned to each mode, *L* is the light transmission distance, *β_i_* is the longitudinal propagation constant of each mode that is related to the equivalent refractive index *n_eff_* of each mode, *Δp* is external random perturbation to each mode, and *N* is the number of modes.

**Figure 9:**
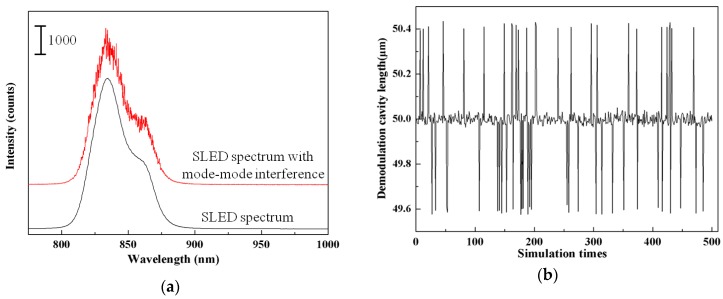
(**a**) Output of SM-MM SLED spectrum and SM SLED Spectrum, (**b**) the 500-times simulation result of demodulated F-P cavity length with random mode-mode interference.

The output spectra of the SLED with SM fiber coupling and with SM-MM fiber coupling are shown in Figure 9a, where the random glitches on the spectrum of SM-MM coupled SLED are caused by the unstable interference field. Meanwhile, the cavity length was demodulated 500 times with a random mode-mode interference in the simulation, shown in Figure 9b. A random error of 0.4 μm of F-P cavity length is induced by mode-mode interference, which leads to the reduction of the demodulation precision. Therefore, in order to reduce the random demodulation errors of the F-P cavity, the fluctuation of SLED spectrum caused by mode-mode interference compared should be as small as possible. We will solve this problem in Section 4.1.

## 4. Experiment and Calibration

### 4.1. Solution to Fiber Mode-Mode Interference

**Figure 10:**
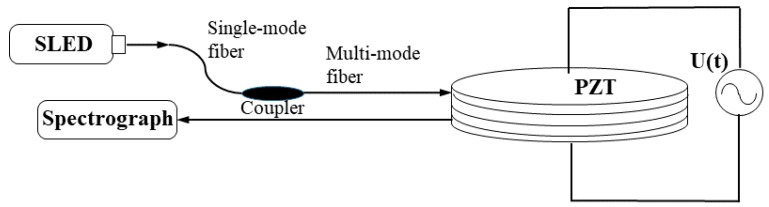
Schematic of a fiber phase modulator based on the piezoelectric ceramic transducer.

We adopted a random phase modulation method, using a piezoelectric ceramic transducer to induce a phase difference on the MM fiber [15]. The schematic figure is shown in Figure 10.

**Figure 11:**
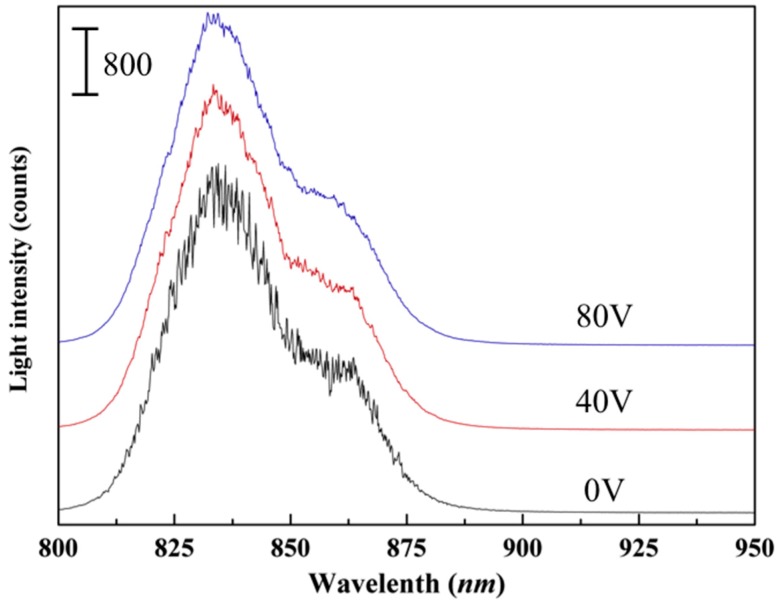
The experimental output of spectrum with the driven voltage of 0 V, 40 V, and 80 V.

According to a number of experiments, a rectangular wave voltage with the frequency of 29 kHz was applied to the piezoelectric ceramic transducer (PZT). The experimental output spectrum was shown in Figure 11, with PZT driven voltage of 0 V, 40 V, and 80 V, respectively. The SLED spectrum with Gaussian distribution became more stable with the increase of the driven voltage.

Here, a parameter root mean squared error (RMSE) is calculated to evaluate the stability of output, which is given by

(5) $$RMSE=\sqrt{\frac{1}{N}\sum_{n=1}^{N}{\left(X_{obs,n}-X_{\mathrm{mod}el,n}\right)}^{2}},$$

where *N* is the number of measurement points in the spectrum, *X_obs,n_* is the light intensity demodulated by PZT, and *X_model,n_* is the light intensity of the SLED spectrum.

**Figure 12:**
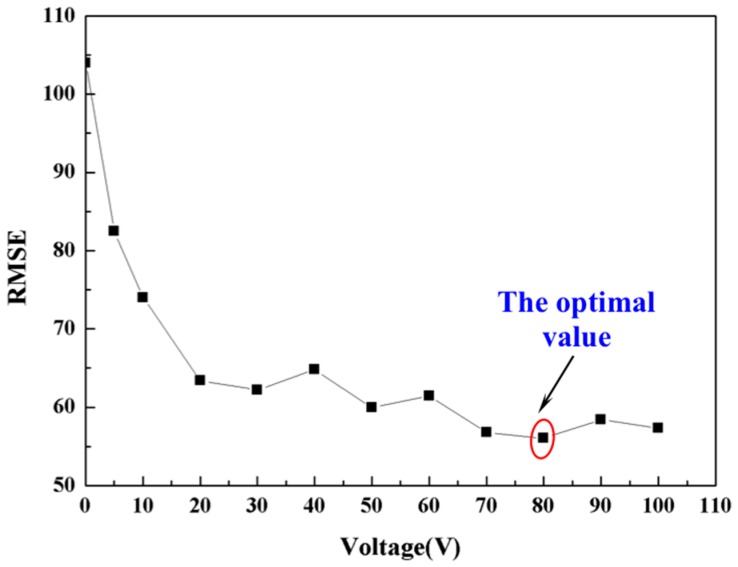
The root mean squared error (RMSE) value at different driven voltage to piezoelectric ceramic transducer (PZT).

The RMSE value tended to decrease with the increase of the driven voltage, and the smallest RMSE value was obtained at the voltage of 80 V, shown in Figure 12. Meanwhile, taking consideration of a driven circuit, 80 V is chosen as the PZT driven voltage in our non-scanning correlation demodulation system.

### 4.2. Calibration of Optical System

As mentioned in Section 2, the pixel position of the peak-intensity in the CCD is used to determine the corresponding thickness of the optical wedge, thus to interrogate the cavity length of the F-P sensor. In fact, the thickness of optical wedge varies linearly with its length, meanwhile the corresponding pixel N of CCD is also linear. Therefore, the demodulation cavity value should be linear with the CCD pixel theoretically. The correspondence between the thickness of optical wedge and the pixel position of peak-intensity in CCD was calibrated.

**Figure 13:**
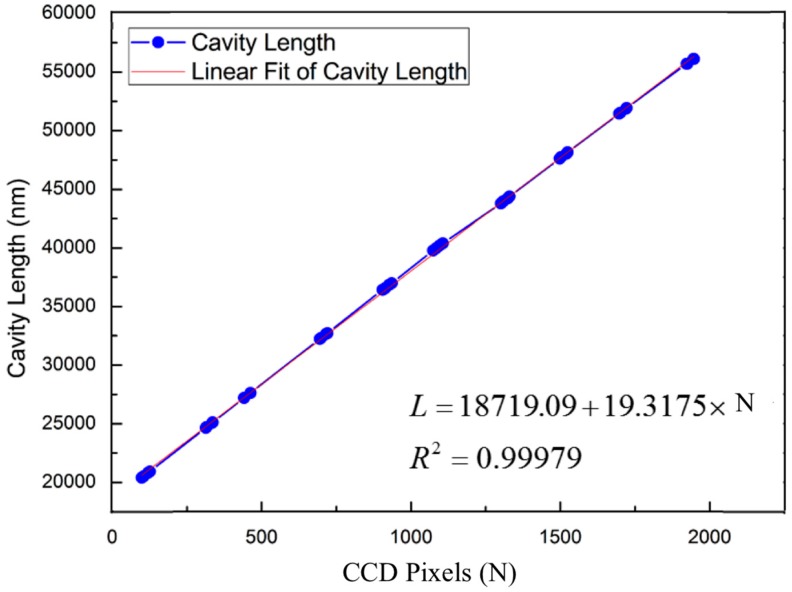
The calibration result of demodulation cavity corresponding to charge coupled device (CCD) pixel.

In the experiment, F-P cavity etalons with standard cavity length (from 20 μm to 55 μm) were demodulated. The corresponding maximum intensity position of CCD pixel was calibrated with the standard F-P cavity length. The result was given in Figure 13, and a very good linearity between CCD pixel position and cavity length was found.

### 4.3. Experiments of the Demodulation System

#### 4.3.1. Dynamic Test of the Demodulation System

**Figure 14:**
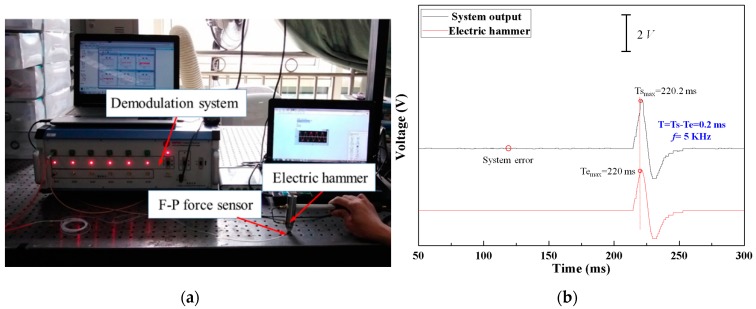
(**a**) The experimental equipment; (**b**) output of electric hammer and the whole demodulation system.

In the experiment, an electric hammer was used to generated a force hitting the F-P force sensor (Figure 14a). In order to investigate the response speed of our demodulation system, the waveform outputs of the electric hammer and the demodulation system were recorded by a high accuracy digital oscilloscope at the exact same time. The demodulation speed of the demodulation system was 5 KHz, according to the analysis of the rising edge (in Figure 14b).

#### 4.3.2. Experiments in the 1:1 Steam Generator Test Loop

**Figure 15:**
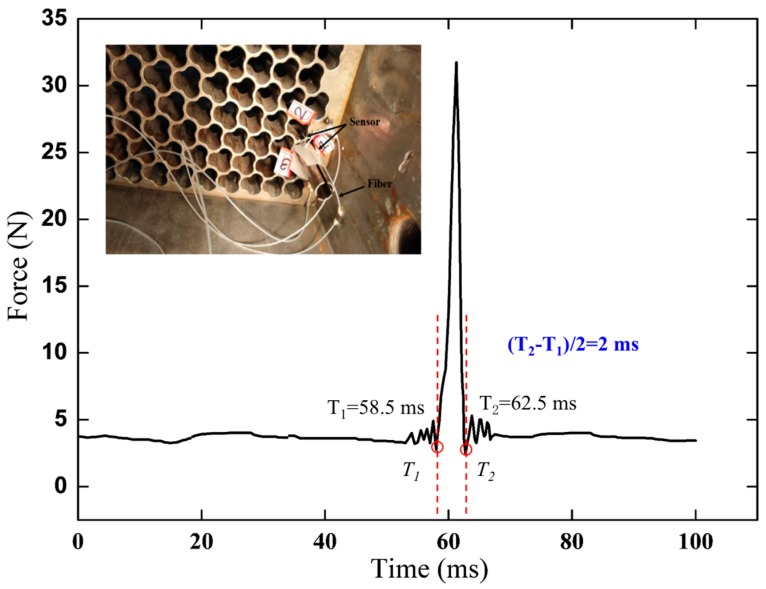
Demodulated force data coming from sensor #1 installed in the SGT, the inset image is the experimental photo when three force sensors installed in a 1:1 steam generator test loop.

With the assistance of our collaborators, the whole demodulation system and force sensor were exploited in a 1:1 steam generator test loop (with the same size, structure, and working conditions as a real steam generator). The fiber F-P force sensors were installed in the contact position between the SGT and TSP, shown in Figure 15 (inset image). The demodulated force data from the SGT were given in Figure 14, manifesting that the collision force is 32 N and the collision time is 2 ms.

## 5. Conclusions

A non-scanning correlation demodulation system was accomplished, capable of processing F-P cavity signal with a demodulation speed of 5 KHz. A clear force signal of about 32 N with the collision time of 2 ms was obtained in a 1:1 steam generator test loop. For the real-time measurement of the radial collision force of SGT, our future work will be in two aspects. First, the stability of our demodulation system will be investigated. Second, a demodulation system with higher demodulation speed and higher accuracy will be studied.

## References

[1] Wade W.C. (1995). Steam Generator Degradation and Its Collision on Continued Operation of Pressurized Water Reactors in the United States. Energy Information Administration/Electric Power Monthly.

[2] Soria S.R., Tolley A., Yawny A. (2017). Characterization of damage and triboparticles resulting from fretting of incoloy 800 steam generator tubes against different materials. Wear.

[3] Jong C.J., Myung J.J., Woong S.K., Young H.C., Hho J.K. (2003). Fretting-wear characteristics of steam generator tubes by foreign object. Nucl. Eng. Technol..

[4] Frick T.M., Sobek T.E., Reavis J.R. (1984). Overview on the Development and Implementation of Methodologies to Compute Vibration and Wear of Steam Generator Tubes. Symposium on Flow-Induced Vibrations, Volume 6: Computational Aspects of Flow-Induced Vibration, Proceedings of the 1984 ASME Winter Annual Meeting, New York, NY, USA, 9–14 December 1984.

[5] Attia M.H., Magel E. (1999). Experimental investigation of long-term fretting wear of multi-span steam generator tubes with u-bend sections. Wear.

[6] Attia H. (2009). A Generalized fretting wear theory. Tribol. Int..

[7] Berthold J.W., Jeffers L.A., Lopushansky R.L. Fiber Optic Sensors for the Refinery of the Future. Proceedings of the ISA/IEEE Sensors for Industry Conference.

[8] Huang P., Wang N., Li J., Zhu Y., Zhang J. (2017). Fiber fabry-perot force sensor with small volume and high performance for assessing fretting damage of steam generator tubes. Sensors.

[9] Hu T., Zhao Y., Li X., Chen J., Lv Z. (2010). Novel optical fiber current sensor based on magnetic fluid. Chin. Opt. Lett..

[10] Ge Y., Wang M., Chen X., Rong H. (2008). An Optical MEMS pressure sensor based on a phase demodulation method. Sens. Actuators A Phys..

[11] Lu E., Ran Z., Peng F., Liu Z., Xu F. (2012). Demodulation of micro fiber-optic Fabry–Perot interferometer using subcarrier and dual-wavelength method. Opt. Commun..

[12] Ran Z., Rao Y., Zhang J., Liu Z., Xu B. (2009). A Miniature fiber-optic refractive-index sensor based on laser-machined fabry–perot interferometer tip. J. Lightwave Technol..

[13] Wang N., Zhu Y., Gong T. (2013). Multichannel fiber optic fabry-perot non-scanning correlation demodulator. Chin. Opt. Lett..

[14] Layton M.R., Bucaro J.A. (1979). Optical fiber acoustic sensor utilizing mode-mode interference. Appl. Opt..

[15] Riedel M. (2003). Piezoelectric Bending Transducer: US. U.S. Patent.

